# Medical News

**Published:** 1852-08

**Authors:** 


*Medical News.*

PROFESSORS Flint and Palmer, of the University of Buffalo. have been appointed to the chairs of *Theory and Practice*, and of *Anatomy*, in the University of Louisville. These chairs were vacated by the resignation of Professors Drake and Cobb, who have returned to the Medical College of Ohio. A. B. Palmer, M. D., of this city, has been appointed to the chair of *Anatomy*, including *General*, *Descriptive*, *Morbid*, and *Microscopic Anatomy*, in the University of Michigan. Drs. F. G. Smith, J. M. Allen and J. J. Reese have accepted appointments to the professorships of *Institutes of Medicine*, *Anatomy*, and *Medical Chemistry* and *Pharmacy*, in the Pennsylvania Medical College.

The chair of Institutes of Medicine is a new creation; all the others have been recently vacated by death or resignation. Dr. Smith is one of the able editors of the *Examiner*. Drs. Allen and Reese have both established reputations as lecturers and teachers in Philadelphia institutions. J.

